# Antimicrobial activity of cell-free supernatant derived from *Ligilactobacillus animalis* SWLA-1 in a novel *ex vivo* canine corneal infection model

**DOI:** 10.3389/fvets.2024.1346313

**Published:** 2024-04-23

**Authors:** Je-Hwan Jang, Hong-Jae Lee, Dong-Hwi Kim, Sang-Won Lee, Joon-Young Kim

**Affiliations:** ^1^Department of Veterinary Ophthalmology, College of Veterinary Medicine, Konkuk University, Seoul, Republic of Korea; ^2^Laboratory of Infectious Diseases and Veterinary Microbiology, College of Veterinary Medicine, Konkuk University, Seoul, Republic of Korea

**Keywords:** bacterial keratitis, multi-drug-resistant staphylococci, *ex vivo* corneal infection, antimicrobial alternatives, corneal ulceration

## Abstract

**Introduction:**

Canine bacterial keratitis is a corneal infection that causes various symptoms, including visual impairment, and necessitates eye removal in severe cases. Staphylococcus pseudintermedius is a pathogen that causes significant bacterial keratitis in canine patients. Moreover, multi-drug resistant Staphylococcus pseudintermedius (MDRSP) has been reported in both humans and animals. Regarding treatment failure against multi-drug resistant (MDR) pathogens with classic antibiotics, antimicrobial compounds derived from probiotics have been suggested as an alternative approach.

**Methods:**

*Ligilactobacillus animalis* SWLA-1 strain and its cell-free supernatant (CFS) have previously demonstrated potent antimicrobial activity against various MDR pathogenic bacteria. Based on this finding, we evaluated the anti-staphylococcal activity of CFS derived from *Ligilactobacillus animalis* SWLA-1 against MDRSP in a newly established ex vivo canine corneal infection model using fresh canine corneoscleral rims. Additionally, an in vitro cytotoxicity test using human keratocytes was performed.

**Results and Discussion:**

CFS significantly inhibited the growth of MDRSP in the novel ex vivo model and did not exhibit any significant toxicity against keratocytes in vitro. Based on these results, the antimicrobial compounds in CFS show potential as a novel approach for MDR staphylococcal keratitis treatment.

## 1 Introduction

Bacterial keratitis is one of the most common type of infectious keratitis in dogs (1), and the major pathogenic bacteria involved in canine corneal ulcers have been isolated and identified. The most prevalent bacterial genera are *Staphylococcus* spp., *Streptococcus*, and *Pseudomonas*. *Staph. pseudintermedius* is the predominant species responsible for canine infectious keratitis in numerous geographical regions (2–7). The emergence of multi-drug resistant (MDR) bacteria, attributed to the misuse and abuse of antimicrobial agents, poses a major global threat to humans and animals (8). In the case of canine bacterial pathogens, MDR *Staph. pseudintermedius* (MDRSP) is a dominant major pathogen with antibiotic resistance (9). As this bacterium is known to be a major pathogen of bacterial keratitis and corneal ulcers in dogs, there have been reported cases of MDRSP infection, including strains belonging to methicillin-resistant *Staph. pseudintermedius* (MRSP) (10–12). Furthermore, many MDRSP strains exhibit resistance to antimicrobial agents used in veterinary ophthalmology, such as fluoroquinolones and tetracyclines (13, 14).

Action plans to combat MDR bacteria and safeguard important antibiotics have been in operation since the late 20th century within international communities. The World Health Organization, Food and Agriculture Organization, and World Organization for Animal Health (formerly known as Office International des Epizooties) have recommended guidelines for the responsible use of antimicrobial agents and listed critically important antibiotics in human and veterinary medicine (15–17). Furthermore, recognizing the need for alternatives to classic antibiotics against MDR bacteria, several proactive studies have suggested discovering and developing novel alternative approaches that safeguard classic antibiotics (18–20). Among them, antimicrobial molecules produced by probiotics, including bacteriocins and organic acids, have been considered promising alternatives to classical antibiotics and an arsenal to fight against MDR bacteria. Various kinds of these molecules have been discovered and reported for their antimicrobial effect (21–23).

**Figure 1:**
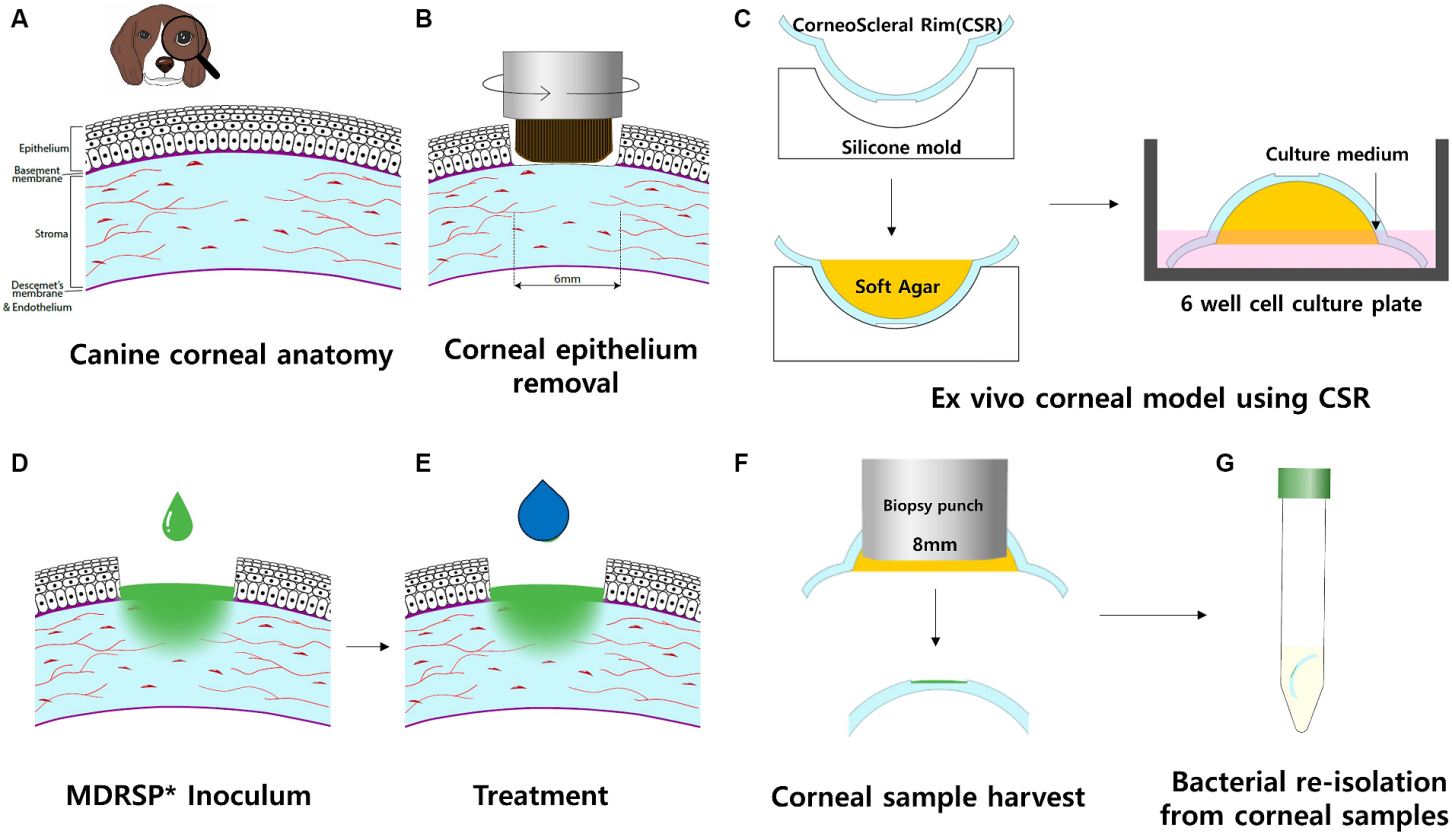
Schematic workflow of the *ex vivo* canine corneal infection model. Each procedure was performed in a sterile environment in a biosafe laminar flow hood. **(A)** Cross-sectional view of the canine cornea, including four layers: epithelium, stroma, Descemet’s membrane, and endothelium. **(B)** Rotating corneal brushes were used to stimulate corneal epithelial removal. **(C)** The corneoscleral rim (CSR) was obtained and placed in a sterile silicone mold with the epithelial side facing down. The agar was then filled with soft agar and left to stand until it solidified. CSRs with solidified agar were placed in a 6-well culture plate with the epithelial side facing up. The culture well was filled with culture medium to the level of the limbus of the CSRs. Culture wells were incubated on a rocking platform. **(D)** Inoculum of MDRSP was applied to the CSR defect sites. **(E)** Thereafter, the same amount of topical treatment was applied per experimental design within a biosafe hood. **(F)** After 48 h of incubation, corneal samples were harvested, and soft agar was removed from each CSR. **(G)** For microbiological analysis, corneal samples were transferred to individual sterile tubes containing 10 mL polybutylene succinate (PBS). *MDRSP: Multi-drug-resistant *Staphylococcus pseudintermedius*.

*Ligilactobacillus animalis* SWLA-1 strain and its cell-free supernatant (CFS), which has versatile antimicrobial activity against both Gram-positive and Gram-negative MDR bacteria, have an inhibitory effect against MDR *Staphylococcus* spp. *in vitro* (24). Based on the findings, we aimed to evaluate the antimicrobial activity of CFS derived from *L. animalis* SWLA-1 against the MDR *Staph. pseudintermedius* KUVM1701GC strain as a potential alternative to commercial ophthalmic antibiotic agents (Figure 1). To simulate the ocular environment resulting from a staphylococcal infection, a new *ex vivo* infection model was established using fresh canine corneoscleral rims (CSRs). In addition, an *in vitro* cytotoxicity assay was performed to determine the cytotoxic effects induced by the CFS derived from *L. animalis* SWLA-1 on live keratocytes.

## 2 Materials and methods

### 2.1 Preparation of canine cornea for *ex vivo* infection model

Canine CSRs were cultured as previously described in *ex vivo* canine models (12). Fresh canine CSRs were obtained from 15 dogs euthanized for reasons unrelated to this study. A total of 30 eyes were extracted from 15 canine subjects. However, six eyes were excluded from the experimental group due to observed corneal pigmentation and degeneration, rendering them unsuitable for the study. Briefly, enucleation of the eye globes was performed by a veterinarian, and no ophthalmic illness was confirmed by macroscopic evaluation. Enucleated globes were immersed in 10% povidone iodine for 30 min at room temperature. Subsequently, the povidone iodine was replaced with sterile phosphate-buffered saline until all visible traces of povidone iodine were eliminated (25). Following this, all globes were transferred to Dulbecco’s modified Eagles’ medium containing 100 μg/mL of streptomycin, 100 units/mL of penicillin, and 0.25 μg/mL of amphotericin. Using slit lamp biomicroscopy, unsuitable samples for this study were excluded from all enucleated eye globes. The corneal culture medium consisted of Dulbecco’s modified Eagle’s medium and Ham’s F-12c nutrient mixture at a ratio of 1:1, supplemented with 10% fetal bovine serum, HEPES buffer, and 0.025% chondroitin sulfate; moreover, the medium was fortified with 10% antimicrobial solution (100 μg/mL of streptomycin, 100 units/mL of penicillin, and 0.25 μg/mL of amphotericin), glutathione, and 1% L-glutamine solution (12).

### 2.2 Preparation of CFS and indicator bacteria

A frozen pure culture of *L. animalis* SWLA-1 was thawed and placed on Difco de Man Rogosa Sharp (MRS) agar (BD Biosciences, Sparks, MD, USA). The plate was incubated at 37°C for 24 h. Five single colonies of *L. animalis* SWLA-1 were selected from the plate, inoculated into the MRS broth, and incubated overnight. This bacterial culture (1.2 × 10^9^ colony-forming unit [CFU]/mL) was collected and centrifuged at 10,000 × g and 4°C for 30 min (Legend X1R; Thermo Fisher Scientific, Waltham, MA, USA) to obtain the crude CFS. As the antimicrobial activity of this CFS was bacteriocin-like, based on a previous study (24), it was concentrated using the trichloroacetic acid (TCA) protein precipitation method. Briefly, 10 mL TCA solution (Sigma-Aldrich, St. Louis, MO, USA) was added to 40 mL crude *L. animalis* SWLA-1-derived CFS. The samples were incubated for 1 h at 4°C for precipitation. The sample was centrifuged at 14,000 × g and 4°C for 10 min. The supernatant was then removed, leaving the protein pellet intact. This pellet was washed with 2 mL of chilled acetone and centrifuged at 14,000 × g and 4°C for 10 min. The washing step was repeated, and the pellet was dried in a 95°C heat block to evaporate any residual acetone. The pure protein pellet was re-dissolved in 50 μL of dimethyl sulfoxide (Sigma-Aldrich, St. Louis, MO, USA) and diluted with 950 μL of Milli-Q water. The protein concentration of the concentrated CFS was quantified using bicinchoninic acid (BCA) protein assay (Pierce™ BCA Protein Assay Kits, Thermo Fisher, USA) and compared with that of the crude CFS. Albumin protein standards were prepared following manufacturer’s recommendation. These protein standards and concentrated CFS were diluted in the working reagent. 25 μL of each sample was mixed with 200 μL of the working reagent at 96-well microliter plate and incubated in 37°C for 30 min. Every mixture was cooled at room temperature and measured the absorbance at OD_562_ on plate reader. Finally, the concentration of CFS was determined using the standard curve, which was generated by plotting blank control and each protein standard. The concentrated CFS samples (5×, 10×, 20×, and 40×) were also evaluated to determine the minimum inhibitory concentration against the indicator bacteria.

The MDRSP strain isolated from a clinical specimen provided by the Konkuk University Veterinary Medical Teaching Hospital (KUVMTH) and designated as *Staph. pseudintermedius* KUVM1701GC was used as the MDR indicator bacterium in this study. This bacterium was collected from the canine patient with chronic endophthalmitis and dermatitis around face. The pure bacterial culture was isolated from eye and skin swab specimen and kept in-80°C. Frozen cultures of *Staph. pseudintermedius* KUVM1701GC were thawed and plated on sheep blood agar. The agar plates were incubated at 37°C for 24 h. Five single colonies of this bacterium were inoculated into tryptic soy broth (TSB; BD, France) and incubated overnight. These bacterial cultures were used in an *ex vivo* model of corneal infection. Using the microdilution method, the antimicrobial susceptibility profiles of the indicator bacteria were evaluated using 15 antimicrobial agents, according to the guidelines of the Clinical Laboratory Standard Institute (CLSI VET01, 2019) and the European Committee on Antimicrobial Susceptibility Testing (EUCAST ver.11.0, 2021).

### 2.3 Evaluation of antimicrobial activity in novel *ex vivo* cornea infection model

To mimic *in vivo* bacterial keratitis, corneal anatomical defenses must be compromised. Using a sterile 6-mm punch biopsy (BIOPSY PUNCH, KAI medical, Japan), a same-diameter defect was created at the center of each cornea. Next, an epithelial brush (Occubrush, Occutech, South Korea) and a rotating brush (Amoils epithelial scrubber; Innovative Excimer Solutions, Inc., Toronto, Canada) were used to consistently remove the corneal epithelium. The corneal epithelium was removed for approximately 10s until no remaining attachments were found in the marking margins. The cornea and scleral rim (5 mm from the limbus) were then excised using a sterile disposable scalpel (15 T, Paragon®, England).

Before preparing the CSR to be placed on the agar base and culture medium, structural support was applied to each CSR to maintain the curvature of the cornea intact. Each cornea was placed on a hemispherical dome silicone mold with a concave-shaped molding surface and filled with low-melting agarose gel (UltraPure LMP Agarose, Thermo Fisher Scientific, Waltham, MA, USA) to prevent excessive heat damage to the cornea. The CSRs were filled with a sterile soft agarose gel and incubated at room temperature for 30 min until the agar solidified. Upon solidification of the agarose solution, the prepared CSRs were placed with the epithelial side facing up on Falcon® 6-well cell culture plates (Corning, NY, USA).

Six prepared CSRs were placed on each 6-well cell culture plate and divided into six groups; (1) Group 1: 20 μL of PBS inoculated on the corneal defect; (2) Group 2: 20 μL *of Staph. pseudintermedius* KUVM1701GC culture inoculated on corneal defect and treated with 20 μL of PBS; (3) Group 3: 20 μL of *Staph. pseudintermedius* KUVM1701GC culture inoculated on corneal defect and treated with 20 μL of vancomycin (20 μg/mL); (4) Group 4: 20 μL of *Staph. pseudintermedius* KUVM1701GC culture inoculated on corneal defect and treated with 20 μL of ofloxacin (3 mg/mL); (5) Group 5: 20 μL of *Staph. pseudintermedius* KUVM1701GC culture inoculated on corneal defect and treated with 20 μL of concentrated CFS (20 × ) derived from *L. animalis* SWLA-1 and (6) Group 6: 20 μL of *Staph. pseudintermedius* KUVM1701GC culture inoculated on corneal defect and treated with 10 μL of concentrated CFS (20 × ) derived from *L. animalis* SWLA-1 and 10 μL of ofloxacin (3 mg/mL). The CSRs belonged to group 1 and group 2 were not treated with any antibiotics in this experiment. Additionally, the CSRs inoculated with indicator bacteria were pre-incubated for 1 h before being treated with PBS, antibiotics or concentrated CFS. A bacterial culture of *Staph. pseudintermedius* KUVM1701GC (OD_600_ = 0.210, 5 × 10^6^ CFU/mL) was used according to the CFU range described by Ubani-Ukoma et al. (25). Since the lowest concentration of concentrated CFS derived from *L. animalis* SWLA-1 that could inhibit indicator bacteria was 10-fold (118.82 ± 3.27 mg/mL), 20-fold concentrated CFS (232.96 ± 5.23 mg/mL) was used in this experiment, with equal volumes of CFS and inoculum applied to the corneal defect. As the indicator bacteria were susceptible to vancomycin and resistant to ofloxacin, these antibiotics were used as reference antibiotics. Treatment with PBS, antibiotics, or concentrated CFS was administered twice a day at 12-h intervals for 48 h. In Group 6, a combination of concentrated CFS and ofloxacin was used to treat the indicator bacteria to determine any synergistic effects between these compounds.

Culture medium was added to each prepared CSR in the well of culture plates until reaching the limbus. The culture plates were placed on a rocking platform shaker, rotating clockwise in three-dimensional axes (20 rotations/min) in a humidified incubator at 37°C with 5% CO_2_ for 48 h.

After 48 h, 8 mm of corneal tissue, including the center of the corneal defect, was aseptically collected from each CSR using a sterile 8-mm punch biopsy (BIOPSY PUNCH, KAI Medical, Japan). The collected corneal tissues were suspended in 10 mL of sterile PBS and serially 10-fold diluted for viable colony counting of *Staph. pseudintermedius* KUVM1701GC. Using the Miles and Misra method, each 20 μL of diluted sample was placed on Staphylococcus No. 110 medium plates (Oxoid, UK). The plates were incubated at 37°C for 24 h. After incubation, the bacterial colonies were counted, and the measured CFU value of each sample was logarithmically transformed (log_10_ CFU/mL) to compare the mean differences in counts between the experimental groups. Experiments were performed independently using four copies of the culture plates.

### 2.4 Corneal opacity analysis

The change in corneal opacity after bacterial infection with CSR in the *ex vivo* corneal infection model was evaluated in each experimental group by three veterinary ophthalmologists. The veterinarians randomly scored pictures of Staphylococcus-negative or Staphylococcus-positive CSR for each experimental group without any information related to the treatments. The scoring of each corneal opacity on macroscopic examination was evaluated using the semiquantitative preclinical ocular toxicology scoring (SPOTS) system (26) and recorded. Briefly, based on this system, the severity of corneal opacity was scored 24 h and 48 h after infection using the SPOTS system (0-normal cornea, 1-minimal loss of corneal transparency, 2-mild loss of corneal transparency, 3-moderate loss of corneal transparency).

### 2.5 *In vitro* cytotoxicity assay

Human keratocytes (ScienCell, Catalog #6520) were obtained and cultured in a fibroblast medium (ScienCell, Catalog #2301). Cultures were maintained at 37°C in an atmosphere with 5% CO_2_.

The cytotoxicity of the concentrated CFS derived from *L. animalis* SWLA-1 (10 × , 118.82 ± 3.27 mg/mL) on human keratocytes was evaluated using Cell Counting Kit-8 assay. Human keratocytes were seeded at a density of 1 × 10^4^ cells per well in a 96-well plate. After 24 h, the cells were treated with serial concentrations of CFS for 72 h. Following the removal of the supernatant, 10% CCK-8 reagent (Sigma-Aldrich, 96,992) in fresh serum-free media was added to the cells. Cells were incubated in a 5% CO_2_ atmosphere at 37°C. After 3 h, a spectrophotometer (Sunrise microplate reader, Tecan, Austria) was used to measure the absorbance of the viable cells at 450 nm. Cytotoxicity was analyzed by comparing the viability of mock-treated cells, which were considered 100% viable controls. This experiment was performed independently in triplicate.

### 2.6 Statistical analysis

The quantitative data in this study were evaluated for normality using the Shapiro–Wilk test. As the mean values of corneal opacity scores and CFU data from corneal samples were not normally distributed, the Kruskal–Wallis test was used to non-parametrically compare the multiple means of the experimental data from each group. This test was used to analyze the differences in the observed mean score or CFU data between the groups. Subsequently, the post-hoc Dunn’s test was used to compare the mean scores and CFU data of each group. Since the observed data were non-parametrically distributed, the Scheirer–Ray–Hare test was also used to analyze the relationships between the mean viable counts of the indicator bacteria and the two variables (experimental groups and corneal opacity scores). Post-hoc Dunn’s test was used to compare the experimental data of each group. All tests were performed using the “rstatix” package [version 0.6.0; R Foundation for Statistical Computing, Vienna, Austria; (27)] and “rcompanion” package [version 2.4.3; Rutgers Cooperative Extension, New Brunswick, New Jersey, USA; (28)] in R (version 4.3.1; R Foundation for Statistical Computing, Vienna, Austria; R core team 2023). Significance was set at an *α* level of 0.05.

## 3 Results

### 3.1 Antimicrobial susceptibility profiles of indicator bacteria

The antimicrobial susceptibility profile of *Staph. pseudintermedius* KUVM1701GC was determined using the microdilution method (Table 1). Among the 15 antimicrobial agents used in this study, the indicator bacteria were resistant to 12 antibiotics (ciprofloxacin, ofloxacin, oxacillin, ampicillin, tetracycline, ceftriaxone, chloramphenicol, gentamicin, azithromycin, trimethoprim/sulfamethoxazole, ceftazidime, and cefotaxime). Only three agents (vancomycin, clindamycin, and amikacin) demonstrated susceptibility to this bacterium. *Staph. pseudintermedius* KUVM1701GC was identified as a methicillin-resistant and MDRSP.

**Table 1:** Antimicrobial susceptibility profile of the *Staphylococcus pseudintermedius* KUVM1701GC strain with the minimum inhibitory concentration.

	MIC (μg/mL)											
Antibiotics	≤0.125	0.25	0.5	1	2	4	8	16	32	64	128≤	Resistance breakpoint (μg/mL)
Ciprofloxacin								16				1
Ofloxacin							8					1
Oxacillin								16				0.5
Ampicillin										64		0.5
Tetracycline										64		1
Vancomycin		0.25										4
Clindamycin					2							4
Ceftriaxone									32			4
Chloramphenicol									32			32
Gentamicin								16				16
Azithromycin									32			2
Amikacin						4						16
Trimethoprim/ Sulfamethoxazole							8/152					4/76
Ceftazidime								16				1
Cefotaxime									32			32

Minimum inhibitory concentrations (MIC) were determined using a 96-well microdilution method according to the Clinical and Laboratory Standards Institute protocol VET01 (CLSI, 2019) and The European Committee on Antimicrobial Susceptibility Testing Version 11.0 (EUCAST, 2021). MIC value of each antimicrobial agent above the resistance breakpoint was present as red and below the breakpoint was present as gray.

The susceptibility of concentrated CFS derived from L. *L. animalis* SWLA-1 against indicator bacteria was also determined using the microdilution method. The minimum inhibitory concentration of CFS was 10-fold concentrated one (118.82 ± 3.27 mg/mL), while the inhibitory effect against *Staph. pseudintermedius* KUVM1791GC was not observed from the CFS concentrated 5-fold (55.74 ± 6.54 mg/mL).

### 3.2 Corneal opacity scoring and microbiological evaluation in *ex vivo* model

**Figure 2:**
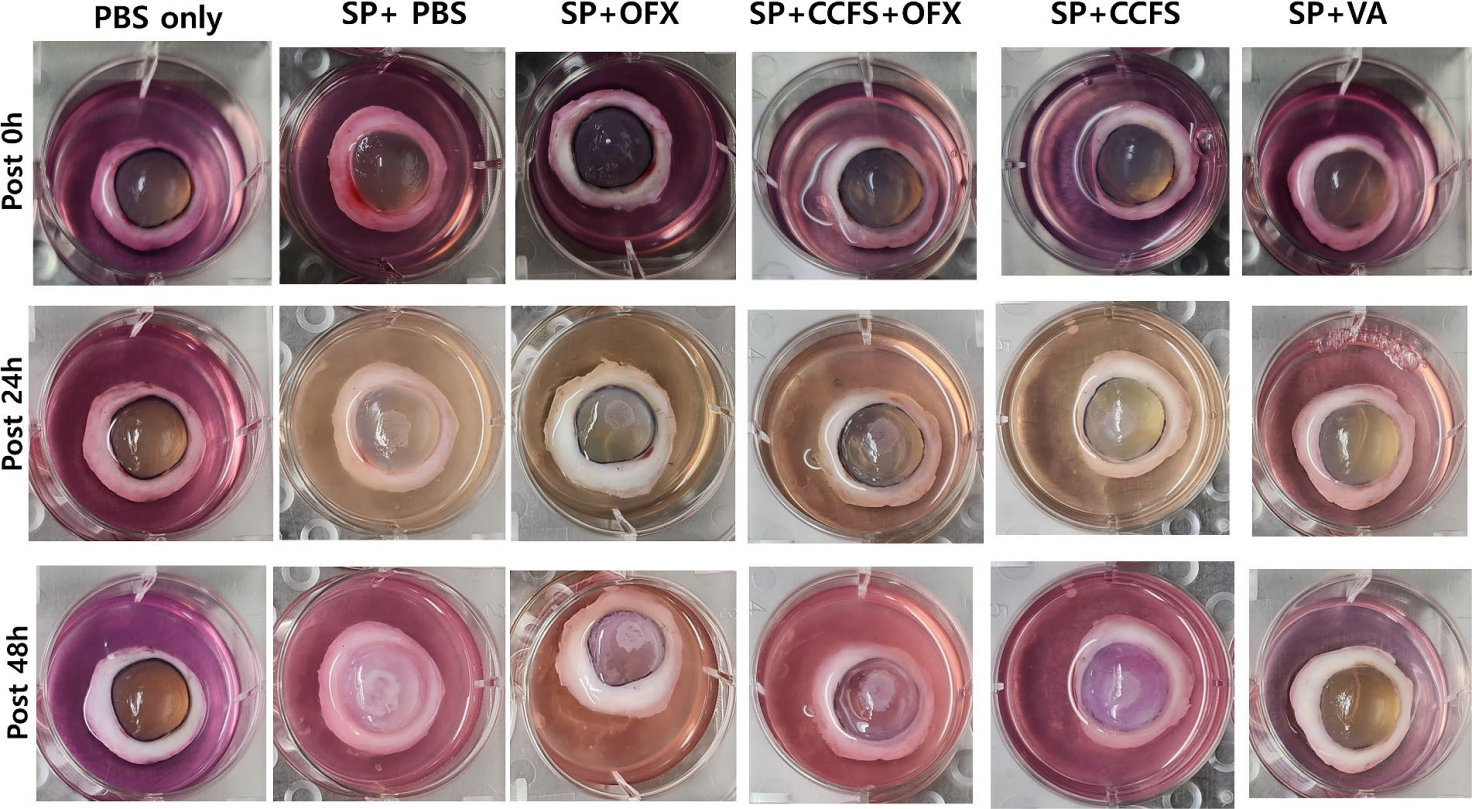
Observation of changes in corneal opacity along the time points. The experimental groups are as follows: PBS only, Staphylococcus-negative and inoculated with sterile PBS only; SP + PBS, Staphylococcus-positive and treated with PBS; SP + OFX, Staphylococcus-positive and treated with ofloxacin (3 mg/mL); SP + CCFS, Staphylococcus-positive and treated with concentrated CFS derived from *L. animalis* SWLA-1 (20×, 232.96 ± 5.23 mg/mL); CCFS+OFX, Staphylococcus-positive and treated with a combination of concentrated CFS (20×, 232.96 ± 5.23 mg/mL) and ofloxacin (3 mg/mL); SP + VA, Staphylococcus-positive and treated with vancomycin (20 μg/mL). PBS: polybutylene succinate.

**Figure 3:**
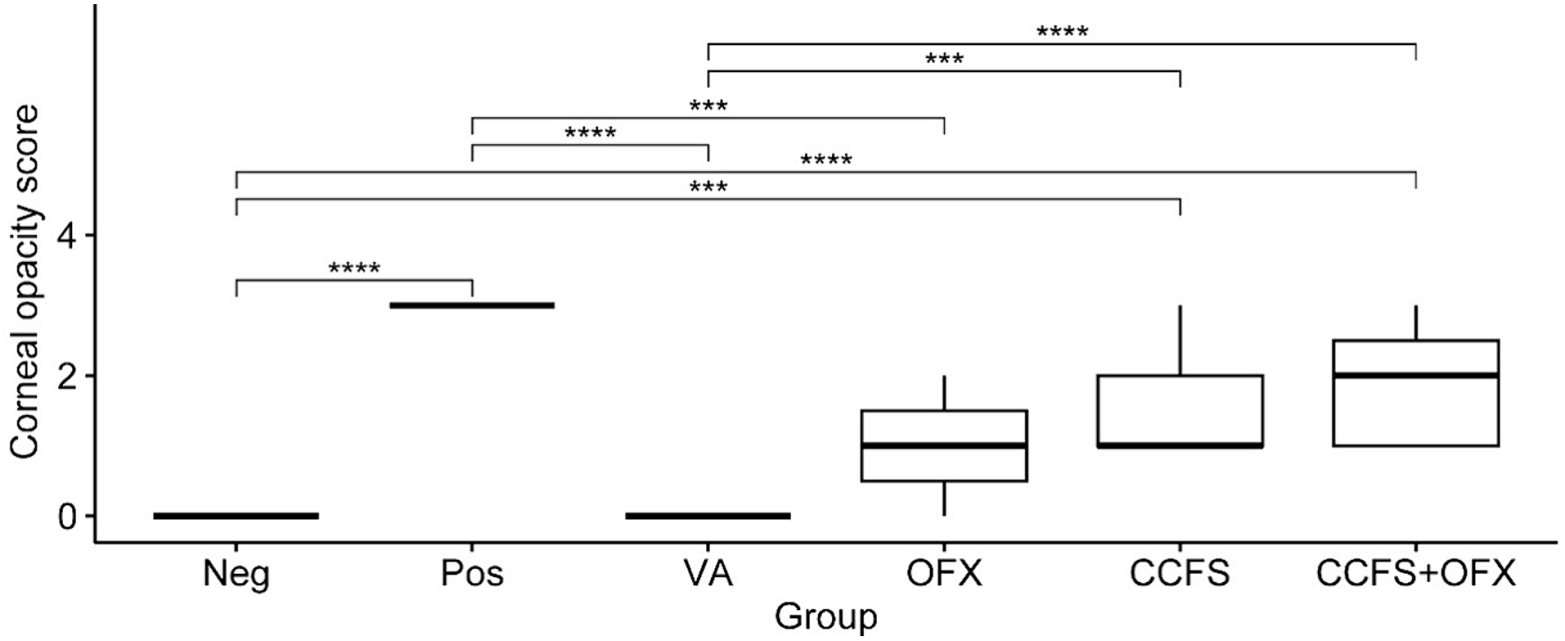
Differences in the mean corneal opacity score between experimental groups. The values of all groups were analyzed using the Kruskal–Wallis test, followed by Dunn’s test for post-hoc testing. The experimental groups are as follows: Neg, Staphylococcus-negative and inoculated with sterile PBS only; Pos, Staphylococcus-positive and treated with PBS; VA, Staphylococcus-positive and treated with vancomycin (20 μg/mL); OFX, Staphylococcus-positive and treated with ofloxacin (3 mg/mL); CCFS, Staphylococcus-positive and treated with concentrated CFS derived from *L. animalis* SWLA-1 (20×, 232.96 ± 5.23 mg/mL); CCFS+OFX, Staphylococcus-positive and treated with a combination of concentrated CFS (20×, 232.96 ± 5.23 mg/mL) and ofloxacin (3 mg/mL). The mean of observed data in each experimental group is presented as the horizontal bold line in the figure. Significant differences are denoted by asterisks (***p < 0.001, ****p < 0.0001).

The changes in corneal opacity after the inoculation of indicator bacteria at each time point (0, 24, and 48 h) are shown in Figure 2. Macroscopic findings showed that corneal opacity increased with time in all experimental groups except for the PBS-only and vancomycin-treated groups. As shown in Figure 3, the mean corneal opacity scores of the groups treated with vancomycin and ofloxacin were significantly lower than those in the Staphylococcus-positive control group (*p* < 0.01). In contrast, the group treated with concentrated CFS or a combination of concentrated CFS and ofloxacin had significantly higher scores than the Staphylococcus-negative control group or vancomycin-treated group (*p* < 0.01). Notably, no significant difference was observed between the ofloxacin-treated group and the CFS or combination of CFS and ofloxacin-treated groups (*p* > 0.05).

**Figure 4:**
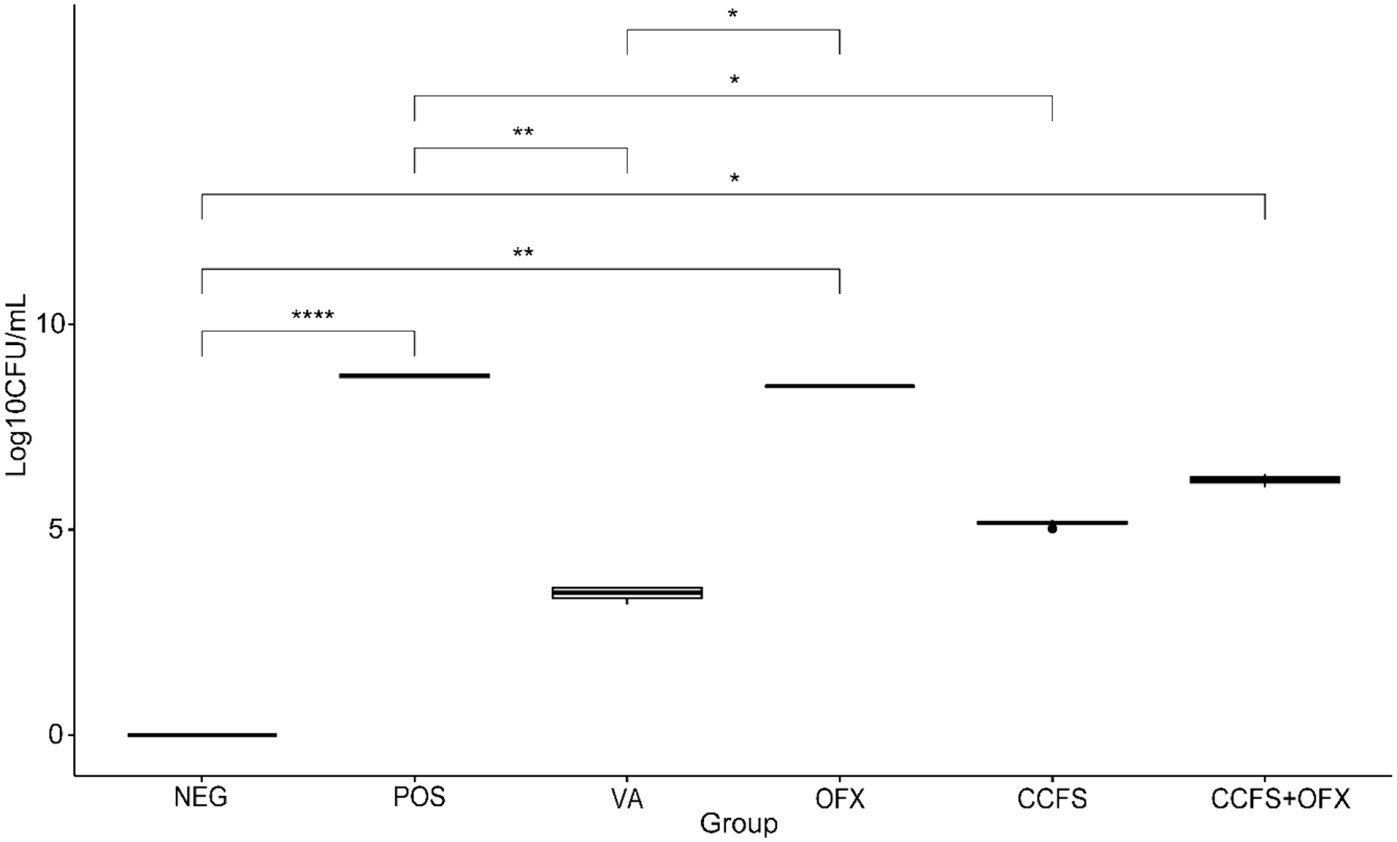
Differences in the mean CFU values between experimental groups. The values of all groups were analyzed using the Kruskal–Wallis test, followed by Dunn’s test for post-hoc testing. The experimental groups are as follows: Neg, Staphylococcus-negative and inoculated with sterile PBS only; Pos, Staphylococcus-positive and treated with PBS; VA, Staphylococcus-positive and treated with vancomycin (20 μg/mL); OFX, Staphylococcus-positive and treated with ofloxacin (3 mg/mL); CCFS, Staphylococcus-positive and treated with concentrated CFS derived from *L. animalis* SWLA-1 (20×, 232.96 ± 5.23 mg/mL); CCFS+OFX, Staphylococcus-positive and treated with combination of concentrated CFS (20×, 232.96 ± 5.23 mg/mL) and ofloxacin (3 mg/mL). The outliers are represented by bullets, and the mean of observed data in each experimental group is represented by the horizontal bold line in the figure. Significant differences are denoted by asterisks (*p < 0.05, **p < 0.01, ****p < 0.0001).

Comparing with the results of the corneal opacity scoring, different results were observed in the microbiological evaluation (Figure 4). The mean CFU values of the group treated with concentrated CFS derived from *L. animalis* SWLA-1 or a combination of concentrated CFS and ofloxacin were significantly lower than those of the Staphylococcus-positive control group (*p* < 0.05). Conversely, the mean CFU value of the ofloxacin-only treated group was significantly higher than that of the Staphylococcus-negative control group. Notably, the growth of *Staph. pseudintermedius* KUVM1701GC was significantly inhibited in the group treated with vancomycin or concentrated CFS compared with that in the other groups, based on the mean CFU differences (*p* < 0.05). Regarding the result of this experiment, the mean CFU values were the only quantitative result showing significant difference between Staphylococcus-positive group and other groups, because the Scheirer–Ray–Hare test results showed no significant interaction between categorical (experimental groups, corneal score) and quantitative (CFU result) variables (*p* > 0.05).

### 3.3 *In vitro* evaluation of cytotoxicity

**Figure 5:**
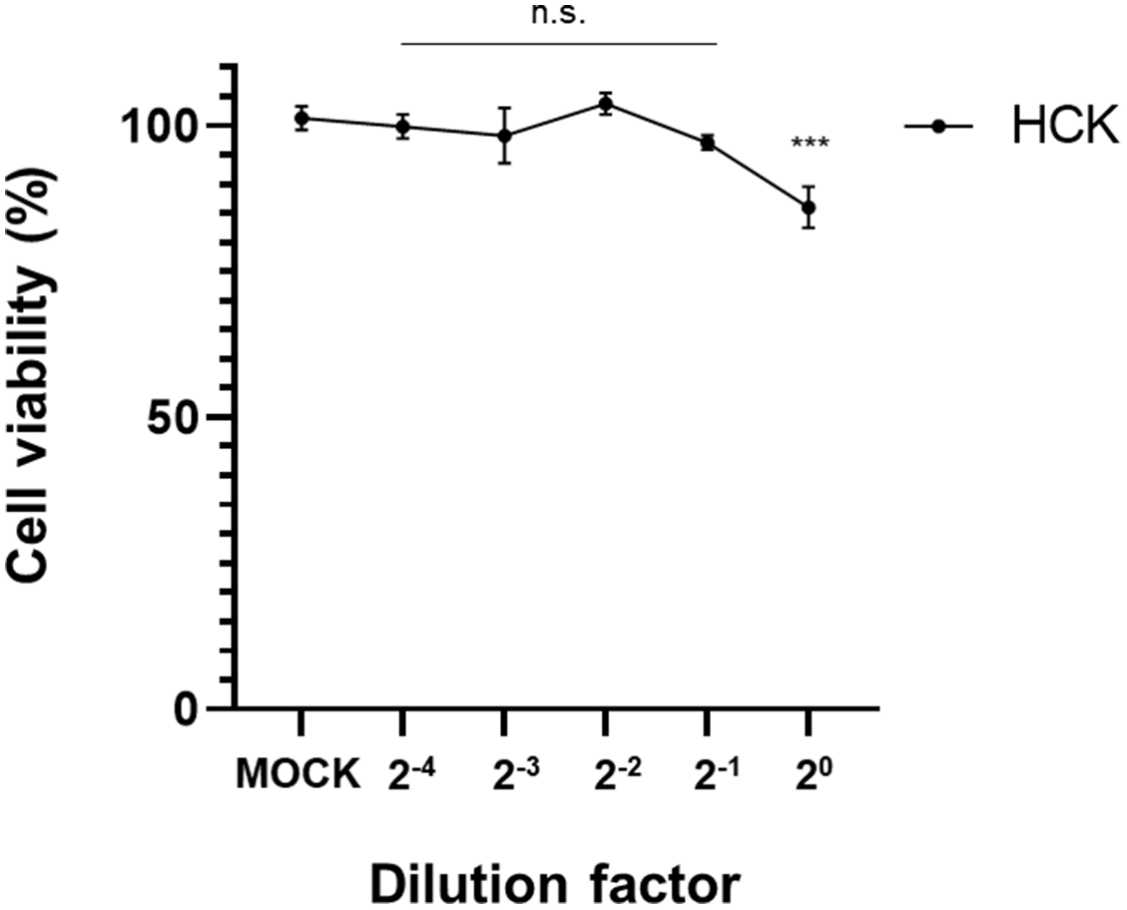
Confirmation of cell viabilities of human keratocytes using the CCK-8 assay. Results were obtained 72 h post-treatment with CFS derived from *L. animalis* SWLA-1, as compared to mock-treated cells at the indicated dilutions. Data are presented as mean ± standard deviation for three independent experiments. Data were analyzed by one-way analysis of variance (n.s: non-significant, ***p<0.001).

The CCK-8 assay demonstrated a mild cytotoxic effect in human keratocytes when exposed to CFS derived from *L. animalis* SWLA-1 compared with that in the mock-treated group. However, after a 2-fold dilution, no significant cytotoxic effects on the viability of human keratocytes were observed (Figure 5). This demonstrated that the 2-fold dilution of CFS did not adversely affect cell viability.

## 4 Discussion

Ulcerative keratitis is one of the most common ocular diseases affecting dogs. Owing to various factors that interact with and adversely affect the ocular surface, the corneal epithelium can become disrupted, leading to exposure of the underlying corneal stroma. When the anatomical barrier of the cornea is compromised or disrupted, it can lead to the invasion of pathogenic bacteria through this weakened gap. A secondary bacterial infection in the corneal stroma can destroy the organized corneal structure, potentially causing vision impairment and necessitating the removal of the whole eye globe in severe cases. Cytological examination, microbiological culture of corneal samples, and antibiotic susceptibility tests are recommended for the diagnosis and treatment of bacterial keratitis (1).

*Staphylococcus pseudintermedius* is the most common opportunistic pathogen responsible for causing infectious keratitis in regions such as America (7, 29, 30), Europe (31), and Asia (10, 32). It has been reported that *Staphylococcus pseudintermedius* can cause the destruction of corneal tissue by expressing an analogue of protein A, which is produced by *Staph. aureus* and plays a major role in staphylococcal keratitis (33, 34). The destruction and lysis of corneal structures induced by bacterial infection increase corneal opacity with the presence of opaque or cloudy area on the cornea (35). The change in corneal opacity and the measurement of bacterial growth using CFU was determined in this study.

The emergence of antimicrobial resistance poses a significant challenge in veterinary ophthalmology (36). In a recent study on canine bacterial keratitis, nearly half of the isolates from clinical *Staphylococcus* spp. infections with corneal stromal ulcers were identified as MRSP. In the same study, one of the isolates from an MRSP-infected patient exhibited multi-drug resistance to antibiotics and presented highly aggressive clinical symptoms (30). Although MDRSP-induced bacterial keratitis is an emerging serious challenge in veterinary medicine, there have been limited studies utilizing corneal infection models of MDRSP. To the best of our knowledge, this is the first study to develop a corneal infection model using MDRSP.

In the present study, a novel *ex vivo* corneal infection model was established to evaluate the antimicrobial activity of CFS derived from *L. animalis* SWLA-1. This model can suggest an alternative approach to evaluate the inhibitory effect of antimicrobial substances CFS derived from probiotic bacteria against MDRSP under simulated conditions similar to bacterial keratitis in live animals. Additionally, owing to the absence of a host immune system in this *ex vivo* model, we successfully evaluated the intrinsic antibacterial properties of CFS derived from *L. animalis* SWLA-1 against the MDRSP KUVM1701GC strain. Furthermore, *ex vivo* corneal infection models are easier to establish and allow for the visible progression of lesions within less than 24 h compared to *in vivo* infection models (37).

We evaluated corneal opacity by scoring corneal haziness 48 h after infection in this study. Clinical features of canine bacterial keratitis include corneal opacity (1, 38). Our results showed no significant correlation between corneal opacity and CFU value among the groups. For instance, the experimental group treated with ofloxacin against indicator bacteria showed significantly lower corneal opacity score compared to the group infected with indicator bacteria and treated with PBS only. However, no significant difference was observed between these two groups based on the result of measuring viable bacterial cell counts. In contrast, the group treated with concentrated CFS showed significantly increased corneal opacity score compared to that of Staphylococcus-negative group. Interestingly, this group was the only one that significantly inhibited the growth of indicator bacteria as well as the group treated with vancomycin, which have no change in corneal opacity and significantly inhibitory effect against indicator bacteria based on viable cell counts. These findings suggest that the clinical assessment of corneal inflammation progression may differ from the actual degree of microbial infection. Further studies should include histopathological analysis of an *in vivo* model of MDRSP infection.

Obviously, *in vivo* corneal models offer an excellent platform for investigating host immune defense, inflammation, and corneal healing mechanisms. Nevertheless, these models are unsuitable for examining the initial stages of infection because of the challenge of infecting healthy, intact corneas. Moreover, the process of infection initiation and progression spans several days and lacks certainty (39). Furthermore, the practical use of *in vivo* studies is hindered by the high cost associated with animal purchase and maintenance costs, as well as ethical concerns regarding the use of dogs as experimental animals.

Recently, there has been considerable interest in utilizing *ex vivo* corneal models to study keratitis. *Ex vivo* tissue models that closely mimic both the biochemical and biophysical aspects are more valuable in terms of efficiency and cost-effectiveness. Various techniques have been reported in the literature to induce bacterial infections in *ex vivo* corneal models, including the use of infected contact lenses (40), corneal scarification (41), and intrastromal injection (37). In this study, we used a rotating epithelial brush to remove the corneal epithelium and establish compromised anatomic defenses (42). This device has been developed for precise corneal epithelium removal during refractive surgery in human medicine, including photorefractive keratectomy and laser-assisted subepithelial keratectomy. Its application ensures the maintenance of consistent size and depth of the corneal defects. Since the *ex vivo* corneal model can also be used to investigate drug delivery systems related to eye infections (25), it can be used in the evaluation of other antimicrobial compounds in further studies.

Probiotics and their antimicrobial substances have been successfully used to prevent and treat various bacterial infections in both humans and animals (43–46). Recently, effective antimicrobial compounds or metabolites derived from probiotic bacteria have been investigated as alternatives to classical antibiotics for treating MDR bacterial infections. These antimicrobial compounds, also known as postbiotics or pharmaceuticals, can contribute to the preservation of classic antibiotics and the treatment of MDR pathogenic bacteria (47). Additionally, antimicrobial compounds derived from probiotics, such as bacteriocins, have significant advantages over classical antibiotics, including reducing the likelihood of inducing antibiotic resistance in bacteria and the relative ease of modification and bio-engineering owing to their molecular size and structures (21, 48).

Based on the results of the microbiological evaluation, the concentrated CFS derived from *L. animalis* SWLA-1 significantly inhibited the growth of MDRSP, comparable to the group treated with vancomycin (*p* < 0.05). As this compound exhibits enhanced antimicrobial activity against pathogens when concentrated using TCA protein precipitation, it appears to function in a concentration-dependent manner, similarly to bacteriocins (49, 50). Further studies should involve the identification and isolation of the active antimicrobial compounds in this CFS through complete genome analysis of *L. animalis* SWLA-1 or peptides and chemical analysis using mass spectrometry.

According to these results, the antimicrobial activity of concentrated CFS derived from *L. animalis* SWLA-1 effectively inhibited the growth of MDRSP in an *ex vivo* corneal infection model. Considering that antimicrobial compounds derived from probiotics have a lower propensity to develop antibiotic resistance in pathogens and are generally recognized as safe for humans and animals, this compound has potential as an alternative to topical antibiotic agents for treating bacterial keratitis.

Although our *ex vivo* infection model proves useful for assessing efficacy, it has limitations. The primary objective of this study was to establish an MDRSP *ex vivo* corneal infection model and assess the efficacy of concentrated CFS derived from *L. animalis* SWLA-1, serving as a preliminary bridge experiment before advancing to an *in vivo* infection model. In contrast to the *in vivo* model, the *ex vivo* model in this study allowed for the evaluation of experimental outcomes with clear macroscopic differences within a short time frame. However, for future experiments utilizing the *in vivo* model, aspects not addressed in this study, such as the interaction between the complete immune system including tear films and bacterial infection over an extended period, will be studied. This will involve a more in-depth investigation through histopathological studies.

Another limitation of this study is the utilization of the SPOTS system’s corneal opacity scoring method in an *ex vivo* environment. SPOTS system was developed to scoring lesions *in vivo* clinically. To better replicate clinical *in vivo* infection conditions, we customized an “air-liquid” culture environment with a rocking platform that stimulates the blinking of the eye. Also, a serum-containing medium were used to supply growth factors and nutrients that replicate *in vivo* conditions following methodology used in previous studies (51, 52). Therefore, considering the *ex vivo* culture setting in this study closely resembles the *in vivo* environment, and with the additional factor of a relatively short culture time of 48 h, we determined that the SPOTS system is suitable for measuring corneal opacity induced by bacterial infection-induced corneal ulceration, which was the focus of our experiment. In future studies, a thorough analysis using anterior segment optical coherence tomography and histopathological studies will be necessary.

This is the first study to describe the establishment of an *ex vivo* MDRSP infection corneal culture model using canine corneas. Furthermore, we confirmed the efficacy of the concentrated CFS derived from *L. animalis* SWLA-1 as a potential alternative antibiotic agent against MDRSP. In future studies, the *ex vivo* corneal infection model can emerge as a robust platform for the comprehensive evaluation of diverse infectious microorganisms and topical therapeutic interventions applicable to both canine and human ulcerative keratitis.

## Data availability statement

The original contributions presented in the study are included in the article/Supplementary material, further inquiries can be directed to the corresponding author.

## Ethics statement

The requirement of ethical approval was waived by Institutinal Animal Care and Use Committee (IACUC) of Konkuk university for the studies involving animals because the tissues were acquired from postmortem conditions; No involvement of animal suffering or manipulation. The studies were conducted in accordance with the local legislation and institutional requirements. Written informed consent was obtained from the owners for the participation of their animals in this study.

## Author contributions

J-HJ: Conceptualization, Investigation, Writing – original draft. H-JL: Formal analysis, Investigation, Writing – original draft. D-HK: Investigation, Writing – original draft. S-WL: Project administration, Writing – review & editing. J-YK: Funding acquisition, Project administration, Supervision, Writing – review & editing.
