# Supplementary material for: Antimicrobial activity of cell-free supernatant derived from Ligilactobacillus animalis SWLA-1 in a novel ex vivo canine corneal infection model
Source: Front Vet Sci. 2024 Apr 23;11:1346313. doi: 10.3389/fvets.2024.1346313 (PMC11074459; doi:10.3389/fvets.2024.1346313)
Supplement: Supplementary file 1 [file Table_1.DOCX]

**Table S1.** **Corneal opacity scoring according to the SPOTS system post 24 hours of infection.**

| **Post 24h** | **Group 1**  **(n=4)** | **Group 2**  **(n=4)** | **Group 3**  **(n=4)** | **Group 4**  **(n=4)** | **Group 5**  **(n=4)** | **Group 6**  **(n=4)** |
| --- | --- | --- | --- | --- | --- | --- |
| **Observer 1** | 0,0,0,0 | 1,0,0,1 | 0,0,0,0 | 1,0,0,0 | 1,0,1,1 | 0,1,0,0 |
| **Observer 2** | 0,0,0,0 | 1,0,0,2 | 0,0,0,0 | 1,1,0,0 | 1,0,1,1 | 1,0,0,0 |
| **Observer 3** | 0,0,0,0 | 1,0,0,1 | 0,0,0,0 | 1,1,0,0 | 1,0,1,1 | 1,0,0,0 |

The severity of corneal opacity according to SPOTS system (0- normal cornea, 1- minimal loss of corneal transparency, 2- mild loss of corneal transparency, 3- moderate loss of corneal transparency)
